# 5α-reductase activity in women with polycystic ovary syndrome: a systematic review and meta-analysis

**DOI:** 10.1186/s12958-017-0242-9

**Published:** 2017-03-27

**Authors:** Chuyan Wu, Ke Wei, Zhongli Jiang

**Affiliations:** 1 Department of Rehabilitation Medicine, the First Affiliated Hospital of Nanjing Medical University, Nanjing, China; 2 Medical Service Section, the First Affiliated Hospital of Nanjing Medical University, Nanjing, China

**Keywords:** 5α-Reductase, Polycystic ovary syndrome, Body mass index, Insulin resistance

## Abstract

**Background:**

5α-reductase activity might be important during the development of polycystic ovary syndrome (PCOS). However, the changes of 5α-reductase activity in PCOS subjects and the relationship between 5α-reductase activity and body mass index (BMI), insulin resistance (IR) remain largely unknown.

**Methods:**

We performed a meta-analysis to examine 5α-reductase activity in women with PCOS; exploratory subgroup analyses were also performed.

**Results:**

Five articles (with 356 cases and 236 controls) reporting 5α-reductase activity in patients with PCOS were selected for the meta-analysis. We observed significantly higher ratios of 5αTHF/THF (5α-reduced tetrahydrocortisol to 5β-reduced tetrahydrocortisol) and An/Et (androsteroneto/etiocholanolone) levels, which were used to assess 5α-reductase activity, among the patients with PCOS, [standardized mean differences (SMD) =0.43, 95%confidence intervals (95%CI) =0.25–0.61, *P* < 0.00001; SMD = 0.86, 95% CI = 0.29–1.44, *P* = 0.003]. We observed significant heterogeneity between studies for An/Et (I^2^ = 89% and *P* < 0.00001). According to the group analysis, women with PCOS exhibited increased 5α-reductase activity which was significantly associated with homeostasis model assessment of insulin resistance (HOMA-IR) regardless of obesity.

**Conclusions:**

5α-reductase activity was enhanced in women with PCOS. Increased 5α-reductase activity in patients with PCOS was related to IR.

**Electronic supplementary material:**

The online version of this article (doi:10.1186/s12958-017-0242-9) contains supplementary material, which is available to authorized users.

## Background

Polycystic ovary syndrome (PCOS) is a heterogeneous syndrome that occurs among women of reproductive age. PCOS affects 6–14% of women of reproductive age [1, 2]. This syndrome is characterized by three main clinical features: hyperandrogenism, abnormal anovulation, and polycystic ovary morphology (via ultrasound) [3]. The diagnostic criteria for PCOS follows the National Institute of Health (NIH) criteria in 1992 [4] or the Rotterdam criteria in 2003 [5]. Hyperandrogenism is the critical factor of the pathophysiologic changes and clinical features associated with PCOS. In women with PCOS, excess androgen originates from the ovaries and adrenals [6]. An increased peripheral conversion of testosterone to the most active androgen 5α-dihydrotestosterone (5α-DHT) occurs via 5α-reductase regulation [7, 8]. Long-term administration of androgens can induce visceral obesity and consecutive insulin resistance. Abdominal adiposity, insulin resistance and compensatory hyperinsulinemia could trigger androgenization in women [9].

5α-reductase is a key enzyme that is important to the metabolic processing of androgens and catalyzes the irreversible conversion of both cortisol to dihydrocortisol in the liver and testosterone to 5α-DHT in the skin [10]. Two isoenzymes of 5α-reductase have been described in previous studies. Increased type 1 isoenzyme is observed in skin and liver, and increased type 2 isoenzyme expression is mainly observed in reproductive tissues. The two types of isoenzymes are encoded by 5 alpha-reductase gene type 1 (SRD5A1) and 5 alpha-reductase gene type 2 (SRD5A2), respectively. It has confirmed that variants in SRD5A1 and SRD5A2 are associated with the prevalence of PCOS among lean women [11]. And increased 5α-reductase activity is present in girls who are at risk for developing PCOS in early childhood [12]. Therefore, changes in 5a-reductase activity might be involved in the pathogenesis of PCOS [11], causing abnormal androgen levels, which is the critical factor of this syndrome.

Some studies have reported significantly increased 5α-reductase levels among subjects with PCOS compared with controls [7, 13–16]. Overexpression of 5α-reductase is also observed in granulosa cells and cultured fibroblasts from patients with PCOS [17, 18]. However, conclusions from similar studies differed. Some large population-based studies reported no differences in the metabolic components of 5α-reductase in 24 h urine samples or in 5α-dihydrotestosterone/testosterone ratios between the experimental and control groups [19, 20]. Therefore, the objective of this study was to systematically assess the changes in 5α-reductase activity in subjects with PCOS by combining the results of previous studies. We also investigated the relationship between obesity, IR and 5α-reductase activity via a meta-analysis.

## Materials and methods

This present meta-analysis was performed based on the PRISMA statement (Preferred Reporting Items for Systematic Reviews and Meta-Analyses). (Additional file 1: Table S1) [21] The present literature includes the literature search, selection criteria, quality score assessment, data extraction and statistical analysis. No registered protocol was required.

### Literature search strategy

A database search was conducted in June 2016. The publications were mainly derived from electronic databases, such as PubMed, Embase, and the Cochrane Library. Only English language and human studies were searched. The following MeSHs terms and their combinations were used to search for included studies: “5α-reductase,” “5alpha-reductase,” “5alpha reductase,” “steroid 5 alpha reductase,” “polycystic ovary syndrome,” “polycystic ovary,” “PCOS”. Only studies with human subjects were searched, and we also performed manual searches within all relevant publications. Two reviewers (CW and KW) searched the key words in the electronic databases independently. Disagreements between the two reviewers regarding data abstraction in the selection process were resolved through discussion.

### Inclusion and exclusion criteria

Two investigators selected articles that assessed changes 5α-reductase activity in women with PCOS. Studies were identified according to the following criteria:

1. Original data were presented.
2. The diagnosis criteria for PCOS were the Rotterdam criteria [5] or the NIH criteria [4].
3. Both an experimental and body mass index- (BMI) matched control group were included.

### Exclusion criteria

1. Means and standard deviations (SDs) (and we could not obtain the original data from the authors) were not reported.
2. Studies that measured mRNA levels of 5α-reductase or measured 5α-reductase levels in tissue.
3. Inclusion of subjects receiving medical treatment and oral contraception pills within 3 months before entry into the study.
4. Inclusion of subjects with other diseases.
5. Letters, case reports, and other forms of content.

### Quality score assessment

The Newcastle-Ottawa Scale was used to evaluate the quality of each included study [22] with some modifications. According to the results of the NOS assessment, the scores ranged between 0 and 7. When the NOS score <4, studies were considered “low-quality,” “Medium” quality studies scored ≤5. A quality score >5 was defined as “high” quality.

### Data extraction

The data were retrieved from each selected study by the two investigators (CW and KW) independently. The general information from each study was systematically extracted using a structured data extraction form: first author’s name, published year, country, matched factors, mean BMI, HOMA-IR, diagnostic criteria for PCOS, measurement method, mean ratio of 5α-reduced tetrahydrocortisol to tetrahydrocortisol (5αTHF/THF), mean ratio of androsterone (5α-reduced androgen metabolite) to etiocholanolone (5β-reduced androgen metabolite) (An/Et), which were used to assess the 5α-reductase activity [23], and their standard deviations (SDs). When the ratios were expressed as medians or geometric values, authors of the original research were contacted to obtain the necessary data. The data retrieved by the two investigators were expected to be the same, and any difference was resolved by consensus.

### Statistical analysis

Heterogeneity in the studies eligible for the meta-analysis was considered significant when P_Q_ < 0.1 according to a chi-squared Q test or I^2^ > 50% according to I-squared statistics. We used a random-effects model according to the results of heterogeneity analysis. To understand the relationship between 5α-reductase activity and the characteristics of PCOS, we further used corresponding subgroup analyses, especially when heterogeneity was obvious. When subgroup analyses were performed, we divided the subjects into a normal group (<25 kg/m^2^) and an over-weight group (≥25 kg/m^2^) according to BMI. The index of HOMA-IR was categorized according to the cut-off values for metabolic syndrome (MS): HOMA1-IR > 2.3 [24]. Studies with data of HOMA not reported or not available were categorized as the NR group.

When heterogeneity was significant, we used a sensitivity analysis to determine the stability of the results. We examined the influence of each included study on the results by excluding individual studies one at a time [2, 25]. The meta-analysis was conducted using the statistical software RevMan 5.2.7 (Cochrane Collaboration,http://www.cc-ims.net/RevMan). Subgroup analyses were performed using the statistical software Stata 12.0 (StataCorp, College Station, TX, USA).

## Results

### Literature selection

**Fig. 1:**
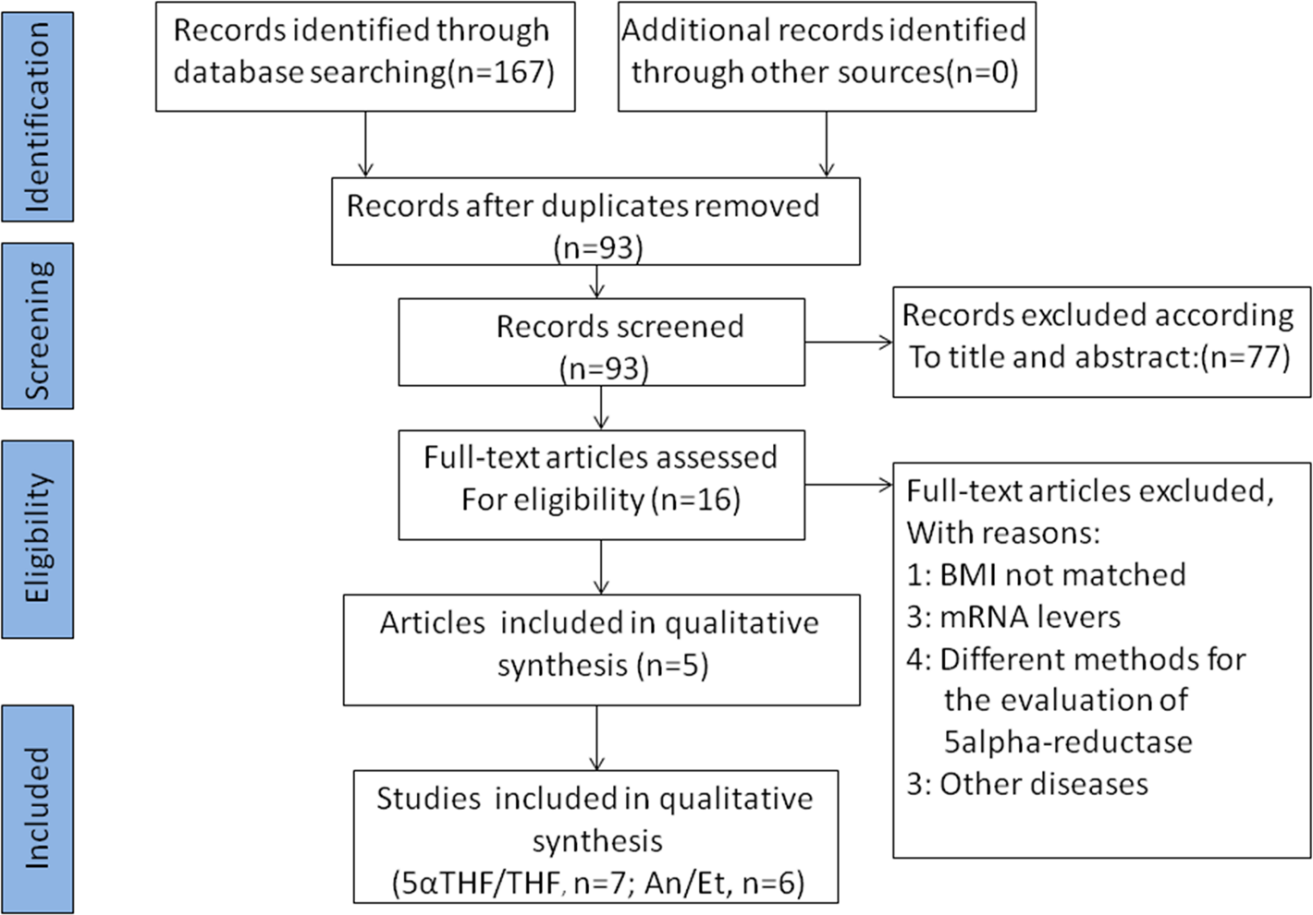
Flow diagram of included studies

Using the outlined search strategy, a total 167 potentially relevant citations were obtained for review. Of the 167 citations, 74 were duplicates, and 77 were not relevant. After removing duplicate and irrelevant articles, 16 studies were selected to be full-text assessed. Of the 16 studies, 11 articles that did not fulfill the selection criteria were excluded (Additional file 2). In two articles, subjects were grouped according to BMI values. One group was the normal group (BMI < 25 kg/m^2^), and the other group was the over-weight group (BMI ≥ 25 kg/m^2^) [15, 19]. Therefore, these two publications were treated as four studies regarding the ratio of 5αTHF/THF. Only one publication was separated into two studies regarding the ratio of An/Et [15]. Finally, 5 articles (*n* = 7 studies) were selected for 5αTHF/THF [13–16, 19], and 5 articles (*n* = 6 studies) were selected for An/Et. Figure 1 shows the flowchart of article selection.

### Characteristics of the studies

The baseline information, matched factors, diagnostic criteria, and general data are clearly shown in the selected studies (Tables 1, 2 and Additional file 3: Table S2). The seven studies eligible for the ratio of 5αTHF/THF included 592 participants (356 PCOS patients and 236 controls). Six case–control studies selected for the ratio of An/Et included the same participants. In three studies, PCOS was diagnosed using the NIH criteria [13, 14, 19], and the remaining two studies utilized the Rotterdam criteria [15, 16]. The PCOS and control groups were matched according to BMI and age. Four studies used urinary gas chromatography/mass spectrometry (GC/MS) analysis to measure cortisol and its metabolites [13–16], and one study used radioimmunoassay to measure cortisol and its metabolites [19]. However, the mean ratios of 5αTHF/THF and An/Et were significantly different across these included studies. Four of the studies reported a higher ratio of 5αTHF/THF in the subjects with PCOS compared with controls. The other three studies reported no difference in enzyme activity. Four of the studies observed higher An/Et ratios in women with PCOS. The other two studies did not observe a difference. In this meta-analysis, two publications (*n* = 3 studies) were defined as medium quality, and the quality scores were 5 [13, 15]. The other three publications (*n* = 4 studies) [14, 16, 19] were considered high quality.

**Table 1:** Characteristics of studies included in the meta-analysis for 5α-THF/THF

Study	Year	Country	Diagnosis Criteria	Npcos	Ncontrol	Matched factors	BMI	HOMA	5α reductase level (5α-THF/THF)	NOS scores
ANDREW RODIN	1994	UK	NIH	34	18	age, BMI	≥25	NR	NS	6
ANDREW RODIN	1994	UK	NIH	31	27	age, BMI	<25	NR	NS	6
D. Chin	2000	USA	NIH	9	29	age, BMI	<25	NR	↑	5
TASOULA TSILCHOROZIDOU	2003	UK	NIH	18	19	age, BMI	<25	1.29	↑	6
Dimitra A. Vassiliadi	2009	UK	Rotterdam criteria	75	28	BMI	≤25	1.3	↑	5
Dimitra A. Vassiliadi	2009	UK	Rotterdam criteria	103	72	BMI	>25	3.1	↑	5
Michael W. O’Reilly	2014	UK	Rotterdam criteria	86	43	age, BMI	>25	2.3	NS	7

*Abbreviations*: *NIH* National Institute of Health criteria, *BMI* body mass index, *NR* no reporting, *NOS* Newcastle-Ottawa Quality Assessment Scale

**Table 2:** Characteristics of studies included in the meta-analysis for An/Et

Study	Year	Country	Diagnosis Criteria	Npcos	Ncontrol	Matched factors	BMI	HOMA	5α- reductase level (An/Et ratio)	NOS scores
ANDREW RODIN	1994	UK	NIH	65	45	age, BMI	≥25	NR	NS	6
D. Chin	2000	USA	NIH	9	29	age, BMI	<25	NR	↑	5
TASOULA TSILCHOROZIDOU	2003	UK	NIH	18	19	age, BMI	<25	1.29	NS	6
Dimitra A. Vassiliadi	2009	UK	Rotterdam criteria	75	28	BMI	<25	1.3	↑	5
Dimitra A. Vassiliadi	2009	UK	Rotterdam criteria	103	72	BMI	≥25	3.1	↑	5
Michael W. O’Reilly	2014	UK	Rotterdam criteria	86	43	age, BMI	≥25	2.3	↑	7

*Abbreviations*: *NIH* National Institute of Health criteria, *BMI* body mass index, *NR* no reporting, *NOS* Newcastle-Ottawa Quality Assessment Scale

### Meta-analysis

**Fig. 2:**
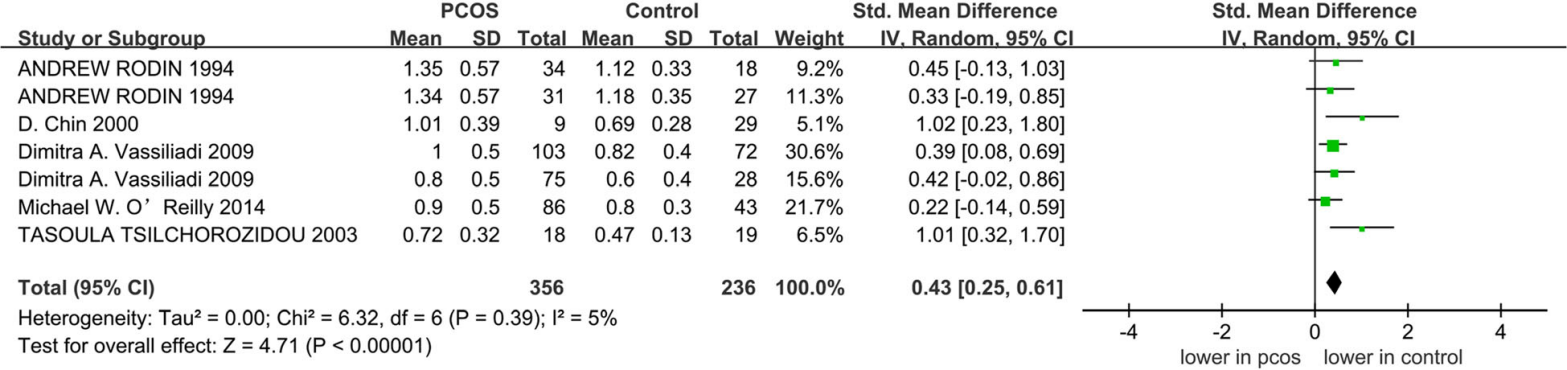
The pooled quantitative synthesis for the ratio of 5αTHF/THF in patients with PCOS compared with controls

**Fig. 3:**
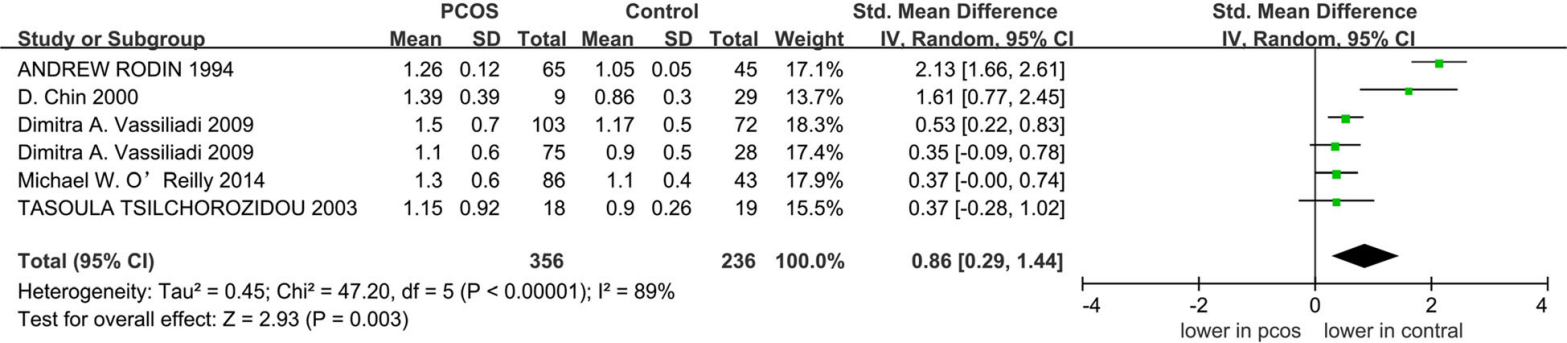
The pooled quantitative synthesis for the ratio of An/Et in patients with PCOS compared with controls

The pooled analysis was performed with the 7 included studies that measured the ratio of 5αTHF/THF. When data from each study were included in the meta-analysis, the ratio of 5αTHF/THF in women with PCOS was significantly higher than the BMI-matched controls (random-effects, SMD = 0.43, 95% CI = 0.25–0.61, *P* < 0.00001; Fig. 2). For the 6 included studies regarding the ratio of An/Et, the patients with PCOS exhibited significantly higher An/Et ratios than the BMI-matched controls (random-effects, SMD = 0.86, 95% CI = 0.29–1.44, *P* = 0.003; Fig. 3). However, significant heterogeneity was observed across the 6 studies eligible for the ratio of An/Et (I^2^ = 89% and *P* < 0.00001).

**Fig. 4:**
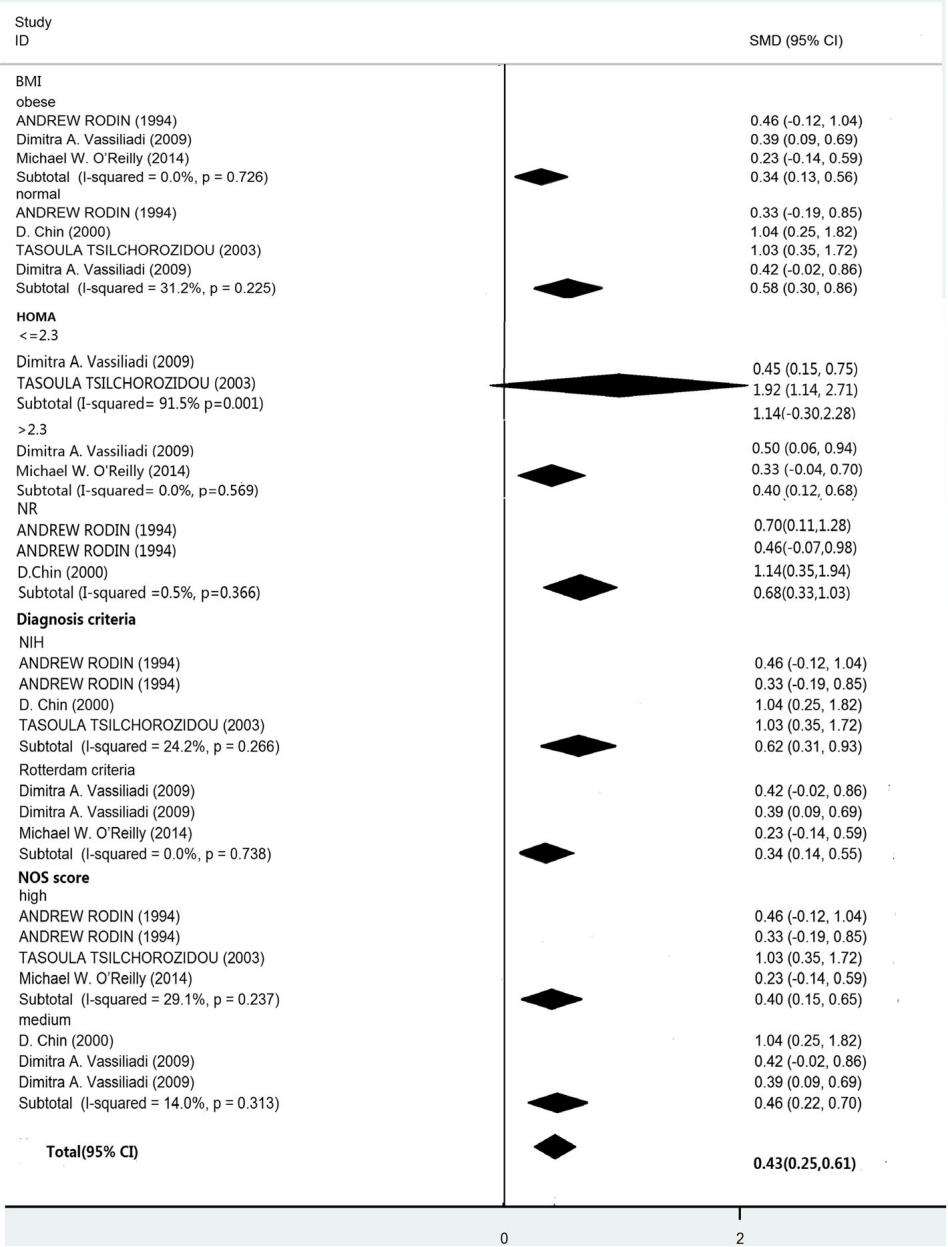
Summary of categorical meta-analysis for 5αTHF/THF

Subgroup analysis: To understand the relationship between obesity, IR and the ratio of 5αTHF/THF in patients with PCOS, subgroup analyses were performed. As shown in Fig. 4, the ratio of 5αTHF/THF in the subjects with PCOS was significantly higher than in controls in all predefined categories for BMI, the diagnostic criteria of PCOS, and NOS scores. Significant differences were also observed both in HOMA-IR group and HOMA-NR group, but not in the group with the value of HOMA < 2.3.

In the subgroup analysis, significant differences in the ratio of An/Et of the subjects PCOS versus controls were found in both the over-weight and normal groups (SMD = 1.00, 95% CI = 0.04–1.96, *P* = 0.042; SMD = 0.72, 95% CI = 0.01–1.43, *P* = 0.047, respectively). There were significant differences in the ratio of An/Et in the quartiles with the diagnostic criteria of the NIH and Rotterdam criteria (SMD = 1.40, 95% CI = 0.26–2.53, *P* = 0.016; SMD = 0.44, 95% CI = 0.23–0.64, *P* = 0.000), also in the quartiles with HOMA-IR and HOMA-NR groups (SMD = 0.46, 95% CI = 0.23–0.70, *P* = 0.517; SMD = 2.02, 95% CI = 1.59–2.45, *P* = 0.306).

**Fig. 5:**
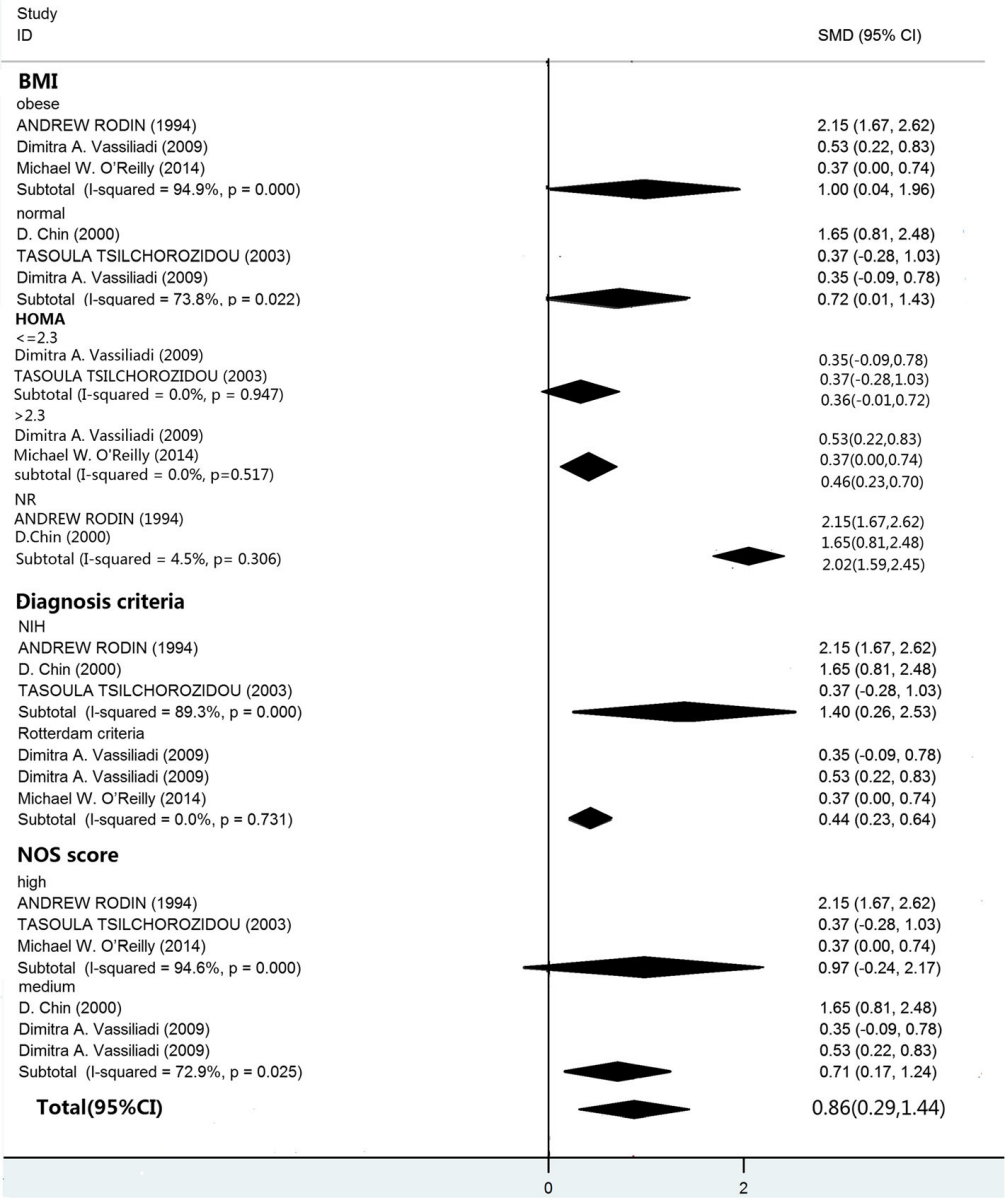
Summary of categorical meta-analysis for An/Et

The subgroup analysis based on NOS scores revealed that significantly higher An/Et ratios were associated with medium-quality studies (SMD = 0.97, 95% CI = −0.24–2.17, *P* = 0.009; I2 = 72.9%, *P* = 0.025 for heterogeneity). However, there was no significant difference in the high-quality score studies (SMD = 0.71, 95% CI = 0.17–1.24, *P* = 0.115; I^2^ = 92%, *P* < 0.001 for heterogeneity; Fig. 5).

### Sensitivity analysis

When studies were omitted one by one, the total SMDs and 95% CIs for both the ratio of 5αTHF/THF and An/Et were not significantly changed. The study by Andrew Rodin et al. [19] utilized a different method measure cortisol and its metabolites. The authors reported impressive negative results whether for 5αTHF/THF or An/Et. When this study was excluded, there were no obvious changes in the results (SMD = 0.48, 95% CI = 0.22–0.73, *P* < 0.001; SMD = 0.53, 95% CI = 0.23–0.82, *P* < 0.001). However, when the study was excluded, the heterogeneity across the 6 studies for the ratio of An/Et decreased significantly (I^2^ = 49% and *P* = 0.10 for heterogeneity).

### Publication bias

Because the number of studies included in this meta-analysis was fewer than 10, we could not use a funnel plot or any other statistical analysis methods to assess the potential publication bias [25, 26].

## Discussion

Although most of the studies had small sample sizes, the activity of 5αTHF/THF and An/Et were significantly higher in subjects with PCOS when compared to BMI-matched controls, and the results from group analysis suggested that high 5αTHF/THF and An/Et levels were significantly associated with HOMA regardless of the degree of obesity or diagnostic criteria. The An/Et ratio between the subjects with PCOS and BMI-matched controls was not significantly different in the “high” quality studies, and there was significant heterogeneity across the 6 studies eligible for the ratio of An/Et.

Recently, pharmacological actions targeting cortisol metabolism as a therapeutic tool have attracted widespread attention. In PCOS women, increased 5α-reductase activity has been associated with idiopathic hirsutism, androgenic alopecia, and acne. Increased 5α-reductase Activity would enhance cortisol metabolism resulting in a compensatory increase in ACTH secretion and stimulation of adrenal steroid-genesis. In women with PCOS, increased 5reductase activity in specific tissues, such as the skin and ovary, has been reported [12]. Even the daughters of women with PCOS have increased 5αTHF/THF ratios [12], suggesting increased global 5α-reductase activity. In the overall analysis, we confirmed the enhanced 5α-reductase activity in women with PCOS. Most patients with PCOS exhibit significant insulin resistance [27] and have changed 5α-reductase activity [7, 28]. 5α-reductase is thought to play an important role in the formation of insulin resistance, which is a major clinical feature of PCOS. The findings of the previous study indicate that enhanced 5α-reductase activity in both men and women is related to insulin resistance [28]. The results of our subgroup analysis also showed that the high levels of 5α-reductase activity in IR groups. In addition, we studied the relationship between increased 5α-reductase activity and obesity. Elevated 5α-reductase activity was observed in both the normal and over-weight groups of women with PCOS, suggesting that enhanced ratios of 5αTHF/THF and An/Et are not associated with obesity. In some previous studies, 5α-reductase was positively correlated with body weight in adult women with PCOS [14, 15]. But one study in 2011 reported genetic alterations in the 5α-reductase gene in lean patients with PCOS, which lead to characteristic clinical changes [11]. Combined with the results of these previous studies, our results from the subgroup analysis suggest that obesity does not affect 5α-reductase activity in patients with PCOS. Because of an extended span of time, our study did not have enough data to perform subgroup analyses of androgen. Further research is required.

It is the first meta-analysis regarding 5α-reductase. Although there were some limitations, our study evaluated the association of the main characteristics of women with PCOS, such as obesity, IR and 5α-reductase activity. Our meta-analysis could provide a direction for future research in this area. In addition, we strictly controlled the inclusion criteria of the articles in this meta-analysis, and we conducted rigorous quality scoring of the literature selected in the study according to NOS criteria. We used various methods to identify potential sources of heterogeneity. During the evaluation of diagnostic criteria for the subgroup analysis, we identified one heterogeneous study. However, the results were not affected after exclusion of this study. Therefore, our results are relatively stable.

There are some limitations that should be considered in this meta-analysis. First, significant heterogeneity was observed in the studies eligible for the ratio of An/Et, and the reliability of our findings might be reduced because of this heterogeneity. The included studies for the ratio of An/Et did not have detailed subgroups (because the heterogeneity was lost in the diagnostic criteria of Rotterdam when the subgroup analysis was performed). PCOS is a kind of endocrine disease with strong clinical heterogeneity. Moreover, the sample sizes in most of the selected studies were not large enough. Because of these influencing factors, it is easy to understand the emergence of significant heterogeneity in our meta-analysis for the An/Et ratio. When we performed the sensitivity analysis, the heterogeneity was significantly decreased after one study was excluded, but the results remained unchanged. We analyzed this study, and the detection methods used for An and Et differed from the other studies, which might be the source for the existence of heterogeneity. However, this study did not cause significant heterogeneity regarding the 5αTHF/THF ratios. Therefore, we did not exclude the study. Second, unpublished data cannot be included, and studies with negative results are difficult to publish. All these factors could lead to the emergence of publication bias in the analysis.

## Conclusions

In this meta-analysis, 5α-reductase activity was enhanced in subjects with PCOS compared with BMI-matched controls, and enhanced 5α-reductase activity in subjects with PCOS was associated with IR rather than obesity. Therefore, increased 5α-reductase activity could be an important biochemical characteristic of PCOS. Given the limited number of studies included in the analysis, the findings from our meta-analysis should be confirmed in future research.

## Additional files

Additional file 1: Table S1. PRISMA 2009 Checklist. (DOC 70 kb)

Additional file 2: The search strategy and excluded studies with reasons. (DOCX 28 kb)

Additional file 3: Table S2. Data from the studies included in the meta-analysis. (DOCX 17 kb)

## Abbreviations

5α-DHT: 5α-dihydrotestosterone
5αTHF/THF: 5α-reduced tetrahydrocortisol to 5β-reduced tetrahydrocortisol
95%CI: 95%confidence intervals
An/Et: Androsteroneto/etiocholanolone
BMI: Body mass index
GC/MS: Gas chromatography/mass spectrometry
HOMA-IR: Homeostasis model assessment of insulin resistance
IR: Insulin resistance
MS: Metabolic syndrome
NIH: National Institute of Health
PCOS: Polycystic ovary syndrome
PRISMA: Preferred Reporting Items for Systematic Reviews and Meta-Analyses
SDs: Standard deviations
SMD: Standardized mean differences
SRD5A1: 5 alpha-reductase gene type 1
SRD5A2: 5 alpha-reductase gene type 2
