# Supplementary material for: 5α-reductase activity in women with polycystic ovary syndrome: a systematic review and meta-analysis
Source: Reprod Biol Endocrinol. 2017 Mar 27;15:21. doi: 10.1186/s12958-017-0242-9 (PMC5369013; doi:10.1186/s12958-017-0242-9)
Supplement: Supplementary file 2 — The search strategy and excluded studies with reasons. (DOCX 28 kb) [file 12958_2017_242_MOESM2_ESM.docx]

Excluded with reasons:

- BMI not matched:[1]
- mRNA levers:[2-4]
- Different methods for the evaluation of 5alpha-reductase:[5-8]
- Other diseases:[9-11]

1. Stewart PM, Shackleton CH, Beastall GH, Edwards CR. 5 alpha-reductase activity in polycystic ovary syndrome. Lancet. 1990;335(8687):431-3. PubMed PMID: 1968168.

2. Jakimiuk AJ, Weitsman SR, Magoffin DA. 5alpha-reductase activity in women with polycystic ovary syndrome. J Clin Endocrinol Metab. 1999;84(7):2414-8. doi: 10.1210/jcem.84.7.5863. PubMed PMID: 10404813.

3. Graupp M, Wehr E, Schweighofer N, Pieber TR, Obermayer-Pietsch B. Association of genetic variants in the two isoforms of 5alpha-reductase, SRD5A1 and SRD5A2, in lean patients with polycystic ovary syndrome. Eur J Obstet Gynecol Reprod Biol. 2011;157(2):175-9. doi: 10.1016/j.ejogrb.2011.03.026. PubMed PMID: 21530059.

4. Wang L, Li S, Zhao A, Tao T, Mao X, Zhang P, et al. The expression of sex steroid synthesis and inactivation enzymes in subcutaneous adipose tissue of PCOS patients. J Steroid Biochem Mol Biol. 2012;132(1-2):120-6. doi: 10.1016/j.jsbmb.2012.02.003. PubMed PMID: 22381227.

5. Fassnacht M, Schlenz N, Schneider SB, Wudy SA, Allolio B, Arlt W. Beyond adrenal and ovarian androgen generation: Increased peripheral 5 alpha-reductase activity in women with polycystic ovary syndrome. J Clin Endocrinol Metab. 2003;88(6):2760-6. doi: 10.1210/jc.2002-021875. PubMed PMID: 12788885.

6. Boda D, Paun D, Diaconeasa A. Evaluation of 5-alpha reductase activity on cultured fibroblast in patients with hyperandrogenemia. Rom J Intern Med. 2009;47(1):67-73. PubMed PMID: 19886072.

7. Magoffin DA. Ovarian enzyme activities in women with polycystic ovary syndrome. Fertil Steril. 2006;86 Suppl 1:S9-S11. doi: 10.1016/j.fertnstert.2006.03.015. PubMed PMID: 16798289.

8. Blumenfeld Z, Kaidar G, Zuckerman-Levin N, Dumin E, Knopf C, Hochberg Z. Cortisol-Metabolizing Enzymes in Polycystic Ovary Syndrome. Clin Med Insights Reprod Health. 2016;10:9-13. doi: 10.4137/CMRH.S35567. PubMed PMID: 27168731; PubMed Central PMCID: PMCPMC4859446.

9. Tomlinson JW, Finney J, Gay C, Hughes BA, Hughes SV, Stewart PM. Impaired glucose tolerance and insulin resistance are associated with increased adipose 11beta-hydroxysteroid dehydrogenase type 1 expression and elevated hepatic 5alpha-reductase activity. Diabetes. 2008;57(10):2652-60. doi: 10.2337/db08-0495. PubMed PMID: 18633104; PubMed Central PMCID: PMCPMC2551674.

10. Baudrand R, Dominguez JM, Carvajal CA, Riquelme A, Campino C, Macchiavello S, et al. Overexpression of hepatic 5alpha-reductase and 11beta-hydroxysteroid dehydrogenase type 1 in visceral adipose tissue is associated with hyperinsulinemia in morbidly obese patients. Metabolism. 2011;60(12):1775-80. doi: 10.1016/j.metabol.2011.05.001. PubMed PMID: 21704348.

11. Torchen LC, Idkowiak J, Fogel NR, O'Neil DM, Shackleton CH, Arlt W, et al. Evidence for Increased 5alpha-Reductase Activity During Early Childhood in Daughters of Women With Polycystic Ovary Syndrome. J Clin Endocrinol Metab. 2016;101(5):2069-75. doi: 10.1210/jc.2015-3926. PubMed PMID: 26990942; PubMed Central PMCID: PMCPMC4870855.
