# Supplementary material for: 5α-reductase activity in women with polycystic ovary syndrome: a systematic review and meta-analysis
Source: Reprod Biol Endocrinol. 2017 Mar 27;15:21. doi: 10.1186/s12958-017-0242-9 (PMC5369013; doi:10.1186/s12958-017-0242-9)
Supplement: Supplementary file 3 — Data from the studies included in the meta-analysis. (DOCX 17 kb) [file 12958_2017_242_MOESM3_ESM.docx]

**Table S2. The data of included studies in meta-analysis**

|  |  | **BMI(kg/m^2^)** | |  | **Age(year)** | |  | **5α-THF/THF** | |
| --- | --- | --- | --- | --- | --- | --- | --- | --- | --- |
| **Study** | **Method** | **PCOS** | **Control** |  | **PCOS** | **Control** |  | **PCOS** | **Control** |

ANDREW RODIN RIA NR NR 25±6 34±5 1.35±0.57 1.12±0.33

ANDREW RODIN RIA NR NR 25±6 31±2 1.34±0.57 1.18±0.35

D. Chin GM 20-36.5 ＜25 14-25 19-45 1.01±0.39 0.69±0.28

TASOULA TSILCHOROZIDOU GM 22.5±2 22.3±1.6 27±4 30±3 0.72±0.32 0.47±0.13

Dimitra A. Vassiliadi GM 25.1±3.1 24.3±3.5 29.0±6.7 35.8±9.6 0.8±0.5 0.6±0.4

Dimitra A. Vassiliadi GM 35.0±4.4 35.2±3.9 30.1±7.4 45.7±8.5 1.0±0.5 0.82±0.4

Michael W. O’Reilly GM 31.9±7.1 30.3±6.4 30.0±7.2 32.4±9.8 0.90±0.5 0.80±0.30

|  |  | **BMI(kg/m^2^)** | |  | **Age(year)** | |  | **An/Et** | |
| --- | --- | --- | --- | --- | --- | --- | --- | --- | --- |
| **Study** | **Method** | **PCOS** | **Control** |  | **PCOS** | **Control** |  | **PCOS** | **Control** |

ANDREW RODIN RIA 24.0-26.3 23.0-27.5 25±6 NR 1.26±0.12 1.05±0.05

D. Chin GM 20-36.5 ＜25 14-25 19-45 1.39±0.39 0.86±0.30

TASOULA TSILCHOROZIDOU GM 22.5±2 22.3±1.6 27±4 30±3 1.15±0.92 0.90±0.26

Dimitra A. Vassiliadi GM 25.1±3.1 24.3±3.5 29.0±6.7 35.8±9.6 1.10±0.60 0.90±0.50

Dimitra A. Vassiliadi GM 35.0±4.4 35.2±3.9 30.1±7.4 45.7±8.5 1.50±0.70 1.17±0.50

Michael W. O’Reilly GM 31.9±7.1 30.3±6.4 30.0±7.2 32.4±9.8 1.30±0.60 1.10±0.40
